# To Treat or Not to Treat: Navigating Early‐Stage CLL in the Era of Targeted Therapy

**DOI:** 10.1111/ejh.70219

**Published:** 2026-05-14

**Authors:** Enrica Antonia Martino, Santino Caserta, Ernesto Vigna, Giovanna Cutrona, Antonella Bruzzese, Francesco Mendicino, Maria Eugenia Alvaro, Caterina Labanca, Eugenio Lucia, Virginia Olivito, Nicola Amodio, Franco Fais, Antonino Neri, Manlio Ferrarini, Valter Gattei, Fortunato Morabito, Massimo Gentile

**Affiliations:** ^1^ Hematology Unit, Department of Onco‐Hematology Cosenza Italy; ^2^ Molecular Pathology Unit IRCCS Ospedale Policlinico San Martino Genoa Italy; ^3^ Department of Experimental and Clinical Medicine University of Catanzaro Catanzaro Italy; ^4^ Department of Experimental Medicine University of Genoa Genoa Italy; ^5^ AIL Sezione di Cosenza Cosenza Italy; ^6^ Scientific Directorate Azienda USL‐IRCCS di Reggio Emilia Reggio Emilia Italy; ^7^ Clinical and Experimental Onco‐Hematology Unit Centro di Riferimento Oncologico di Aviano (CRO) IRCCS Pordenone Italy; ^8^ Department of Pharmacy, Health and Nutritional Science University of Calabria Rende Italy

**Keywords:** chronic lymphocytic leukemia, early‐stage disease, prognostic biomarkers, risk stratification, watch‐and‐wait strategy

## Abstract

Chronic lymphocytic leukemia (CLL) is most frequently diagnosed at early, asymptomatic stages (Rai 0/Binet A), in which a watch‐and‐wait strategy remains the standard of care, based on historical trials demonstrating no overall survival benefit from early treatment. Over the past two decades, however, substantial advances in genomic profiling—including immunoglobulin heavy‐chain variable region (IGHV) mutational status, TP53 disruption, recurrent gene mutations, and complex karyotype—have uncovered marked biological heterogeneity among early‐stage patients and substantially improved prediction of disease progression. In parallel, targeted therapies such as Bruton tyrosine kinase (BTK) inhibitors and venetoclax‐based combinations have transformed the management of symptomatic CLL, raising renewed interest in whether early intervention might favorably alter the natural history of biologically high‐risk disease. In this review, we critically examine the evolution of prognostication in early‐stage CLL, integrate contemporary molecular and clinical risk models, and summarize evidence from both historical chemotherapy‐era studies and modern early‐intervention trials. We discuss key unresolved controversies, including reliance on surrogate endpoints, the risks of overtreatment, and the persistent absence of an overall survival benefit across all early‐treatment strategies. Finally, we outline future research priorities, including refined genomic stratification, minimal residual disease‐driven (MRD)‐driven approaches, and combination targeted therapies currently under investigation. Despite renewed interest in preemptive treatment, available evidence supports continued observation for asymptomatic patients outside clinical trials.

## 1 Introduction

Chronic lymphocytic leukemia (CLL) is the most common leukemia in adults in Western countries [1] and is increasingly diagnosed at early, asymptomatic stages as a consequence of widespread laboratory testing [1]. Although patients classified as Rai 0 or Binet A share similar clinical characteristics—their clinical trajectories are highly heterogeneous [2, 3]. While many patients remain asymptomatic for decades, a substantial subset experiences disease progression within a few years and ultimately requires treatment [4, 5, 6].

In this context, the time to first treatment (TTFT), defined as the time from diagnosis/first presentation to the initiation of the treatment, rigorously established according to the iwCLL guidelines [5], has emerged as a robust and clinically meaningful endpoint, reflecting the CLL biology in a therapy‐independent manner, especially in early‐stage CLL, where overall survival (OS) is often confounded by prolonged disease duration and competing causes of death. A large multicenter study demonstrated that TTFT reliably reflects underlying disease biology and can be effectively predicted using integrated clinical and biological risk models [7]. Several subsequent analyses have validated TTFT as a surrogate marker of progression risk, showing superior prognostic discrimination compared with traditional anatomical staging systems alone [8]. More recently, TTFT has been shown to independently predict OS, reinforcing its value not only as a marker of disease kinetics but also as an integrative determinant of long‐term outcome in CLL [9].

Advances in immunogenetics, cytogenetics, and extensive molecular profiling have profoundly transformed our understanding of CLL pathogenesis and risk stratification. Established prognostic markers—such as immunoglobulin heavy‐chain variable region (IGHV) mutational status, TP53 disruption, complex karyotype, and recurrent mutations—including NOTCH1, SF3B1, ATM, and BIRC3—enable refined prognostic categorization and identify patients at increased risk of early progression [10, 11, 12, 13, 14, 15, 16, 17, 18, 19, 20]. Beyond single lesions, emerging data on genomic complexity, clonal evolution, and chromosomal instability further support the concept that early‐stage CLL comprises biologically distinct entities despite a uniform clinical presentation [21, 22].

Concurrently, the therapeutic landscape of CLL has been transformed by the advent of targeted therapies, including Bruton tyrosine kinase inhibitors (BTKis), BCL2 inhibitors, and next‐generation anti‐CD20 monoclonal antibodies [23, 24, 25]. These therapies achieve deep and durable remissions with a more favorable toxicity profile than traditional chemoimmunotherapy, prompting renewed interest in the potential role of early intervention for biologically high‐risk, asymptomatic patients [26, 27, 28]. However, a central question remains unresolved: does early treatment translate into meaningful improvements in OS or quality of life (QoL), or does it merely anticipate therapy initiation without conferring long‐term benefit? To date, early‐intervention trials have demonstrated improvements in surrogate endpoints but have failed to show a clear OS advantage [29, 30]. Accordingly, current guidelines continue to recommend initiating treatment only when patients develop symptomatic disease [5].

In this review, we integrate current biological and clinical evidence to contextualize the management of early‐stage CLL in the era of targeted therapies. We critically examine contemporary prognostic models, review historical and modern early‐intervention trials, and discuss the unresolved controversies that continue to shape clinical practice. Our objective is to provide an evidence‐based perspective on whether the long‐standing watch‐and‐wait paradigm should be reconsidered in selected patients in light of emerging data from early‐intervention trials with targeted therapies.

## 2 Biologic Stratification of Early‐Stage CLL and Time to First Treatment

**FIGURE 1:**
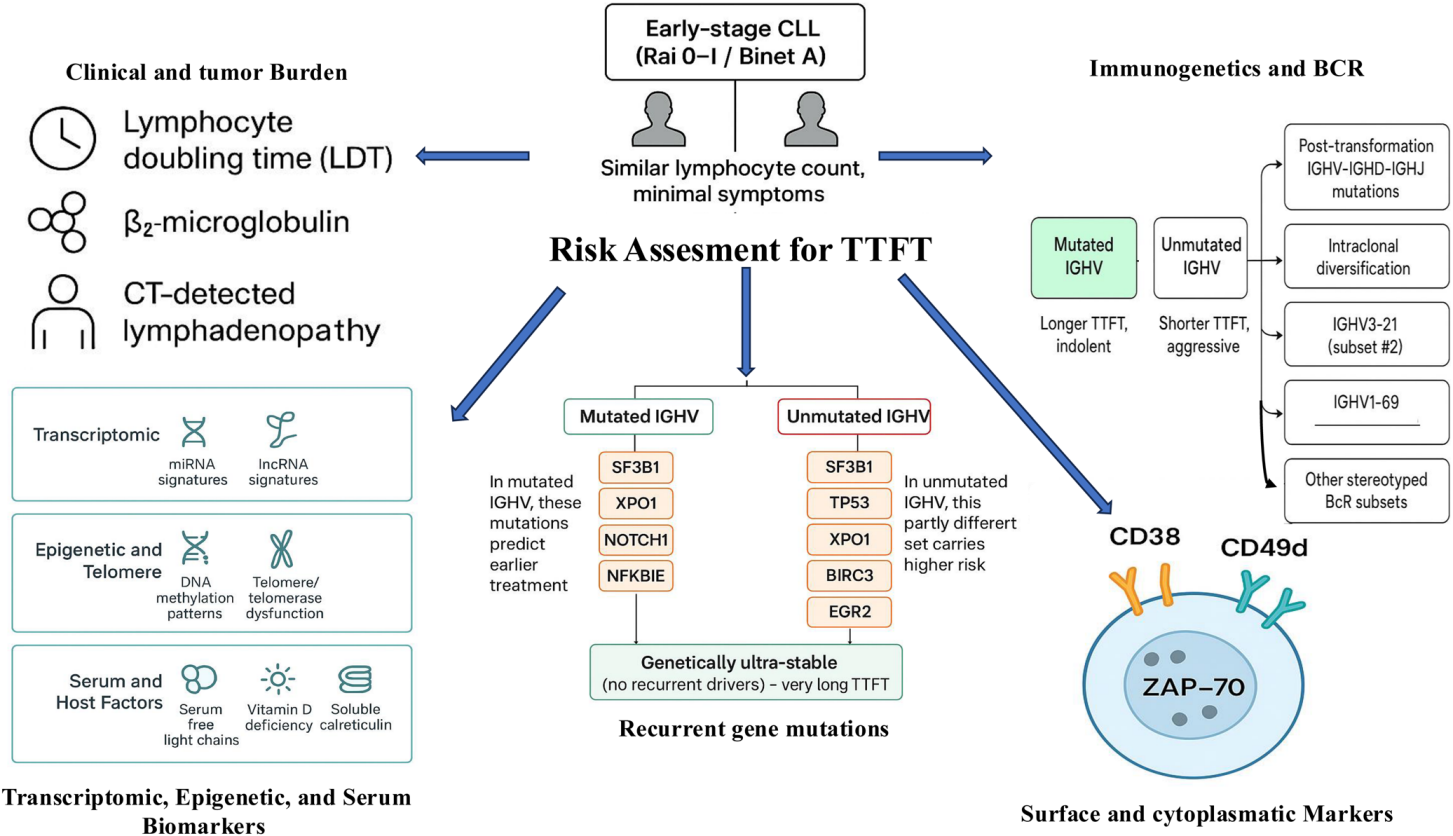
Biological heterogeneity and predictors of time to first treatment (TTFT) in early‐stage CLL. Early‐stage CLL patients with similar Rai/Binet stage and lymphocyte counts can have indolent or progressive courses. Parameters measuring tumor burden (LDT, β2‐microglobulin, CT‐detected lymphadenopathy), IGHV/BcR profile, cytogenetic lesions, recurrent gene mutations, and surface and cytoplasmatic markers define biologically distinct subgroups with differing risk of progression and TTFT.

Clinical staging systems such as Rai and Binet provide a useful framework for initial stratification but fail to capture the profound biological heterogeneity of early‐stage CLL [2, 3]. As illustrated in Figure 1, early‐stage CLL is not a uniform entity: TTFT is driven by multiple, interacting layers of risk. Clinical stage and simple laboratory parameters explain only part of disease variability, whereas immunogenetic features, cytogenetic and mutational architecture, and selected surface, transcriptomic, and serum biomarkers progressively refine prognostication. Integrating these dimensions shifts risk assessment from a purely anatomical approach to a biologically driven, individualized estimation of TTFT, supporting tailored monitoring strategies and the identification of patients who may benefit from closer surveillance or earlier therapeutic interventions [20].

Although lymphocyte doubling time (LDT) and serum β2‐microglobulin (β2‐M) provide relevant prognostic information, their predictive accuracy is limited when used in isolation, and they are insufficient to fully anticipate progression [31, 32, 33].

### 2.1 GHV Status, Mutational Load, and Intraclonal Diversification

Among biological determinants, IGHV mutational status remains the most robust and reproducible prognostic marker in early‐stage CLL [10, 11], useful also in guiding the intensity of patients' follow‐up. Unmutated IGHV is associated with a more rapid evolution of Monoclonal B Lymphocytosis (MBL) to overt CLL [34], with shorter TTFT and a more aggressive disease, whereas mutated IGHV often confers prolonged clinical stability [10, 11]. The additional prognostic value of quantifying IGHV mutational load beyond binary classification has been extensively investigated. In a cohort of more than 1000 patients, it has been demonstrated that mutational load does not outperform conventional mutational status for TTFT prediction, as most unmutated cases lack somatic hypermutation and borderline cases (1%–2%) show similar kinetics [35].

Raponi et al. further clarified the behavior of borderline IGHV cases, confirming that TTFT is primarily driven by binary IGHV status rather than minor gradations in mutational load [36].

Dynamic aspects of IGHV biology further refine risk stratification. Post‐transformation IGHV/IGHD/IGHJ mutations, a phenomenon also known as intraclonal diversification (ID), have been shown to influence disease behavior and progression risk [37]. In this regard, recently, Vit et al., thanks to an algorithm capable of dichotomizing CLL cases into discrete categories with different ID levels, were able to demonstrate that ID may provide additional prognostic information: IGHV mutated CLL with high ID shows longer TTFT than mutated CLL without/with low ID levels, the latter progressing earlier than expected [38].

### 2.2 IGHV Subtypes, BcR Stereotypy, and Risk Scores

Beyond overall mutational status, specific IGHV subtypes and stereotyped B‐cell receptor (BcR) configurations confer distinct prognostic implications. In particular, CLL expressing the IGHV3‐21 in association with a specific CDR3 (the so‐called stereotyped subset #2) is associated with aggressive disease and shorter TTFT independently of IGHV mutational status; IGHV1‐69 has also been linked to adverse outcomes, as well as the expression of the stereotyped subset #1 (IGHV1 family other than IGHV1‐69 in conjunction with a specific CDR3 sequence) [39, 40].

Arguello Tomas et al. evaluated five prognostic scores in 781 Rai 0/Binet A patients and demonstrated that incorporating IGHV subset #2 significantly improves risk stratification beyond conventional models [41].

### 2.3 Cytogenetic Abnormalities and Complex Karyotype

Cytogenetic lesions provide complementary prognostic information in early‐stage CLL. Isolated del(13q14) is associated with a favorable outcome, trisomy 12 with intermediate risk, and del(11q) or del(17p) with aggressive disease and shorter TTFT [12, 21, 22, 42]. Gain of chromosome 2p identifies a subgroup with increased proliferative activity and higher progression risk [43]. Integration of TP53 sequencing with FISH is essential, as subclonal TP53 mutations profoundly affect disease course [21, 22, 44, 45, 46].

Complex karyotype (CK) has emerged as a strong marker of biological aggressiveness. CK (≥ 3 abnormalities), particularly high CK (≥ 5), is associated with significantly shorter TTFT and frequently coexists with TP53 disruption and unmutated IGHV [14, 19, 22]. Importantly, CK retains independent prognostic value after adjustment for major genetic lesions, defining a subgroup with marked “biologic urgency” warranting closer monitoring and consideration for clinical trials [14, 19, 22].

### 2.4 Surface Markers and Flow‐Cytometric Surrogates

Surface markers such as CD38 and ZAP‐70 have been associated with shorter TTFT, although they are not recommended by guidelines because of the lack of harmonization and reproducibility of the assays that are used to test their expression, limiting their clinical applicability [47, 48, 49, 50].

In contrast, CD49d is one of the most robust flow‐cytometric predictors of progression [51], independently associated with reduced TTFT even within otherwise favourable biological subgroups [52]. Collectively, these markers may act as practical surrogates for IGHV status when sequencing is unavailable and predict TTFT in early‐stage disease [53].

### 2.5 Recurrent Gene Mutations and Clonal Architecture

Next‐generation sequencing has revealed that recurrent mutations beyond TP53—including NOTCH1, SF3B1, ATM, BIRC3, XPO1, EGR2, and NFKBIE—significantly affect TTFT, with prognostic impact modulated by IGHV status [54]. NOTCH1 mutations are consistently associated with accelerated disease progression and earlier treatment requirements [13, 55, 56]. Conversely, genetically “ultra‐stable” CLL lacking detectable driver alterations is characterized by remarkably prolonged TTFT [57]. Specific attention should be paid to mutations of the XPO1 gene, which have been associated with a significantly shorter TTFT. Beyond their prognostic value, XPO1 mutations are of particular clinical interest as they represent a potentially druggable target through the use of selective inhibitors of nuclear export (SINE), such as selinexor [58]. Despite their clear biological relevance, technical challenges, variability in sequencing platforms, and lack of standardization currently limit the routine clinical application of mutation‐based models. Indeed, none of these mutations is recognized as a prognosticator by current clinical guidelines, and their use is largely confined to research settings.

### 2.6 Transcriptomic and Epigenetic Biomarkers

Transcriptomic regulators, particularly microRNAs (miRNAs) and long non‐coding RNAs (lncRNAs), add a layer of prognostic information. In the O‐CLL1 cohort, a multicenter, observational study conducted in Italy with the aim of evaluating the diagnosis and management of patients with CLL and related conditions in real‐world clinical practice, it has been shown that a 16‐miRNA signature improves TTFT prediction beyond established clinical and molecular models [59]. Additional biomarkers capture complementary aspects of tumor biology and host–tumor interaction. Telomere/telomerase system impairment [60], global promoter methylation patterns [61], elevated serum free light chains [62], increased BAFF serum concentration [63], vitamin D insufficiency [64], daily intake of polyphenon E and epigallocatechin gallate [65], Whole Food Plant‐Based (WFPB) diet [66], soluble calreticulin [67], and lncRNA signatures [68], tumor mutational load [69] have all been associated with TTFT. Moreover, changes in DNA methylation during normal B‐cell maturation provide valuable information: disease aggressiveness is linked to the degree of epigenetic maturation and transcription‐factor dysregulation [70].

### 2.7 Disease Burden, Imaging, and Classical Clinical Parameters

Baseline disease burden remains relevant. O‐CLL1 studies demonstrated that occult lymphadenopathy detected by total‐body CT in Rai 0/Binet A patients predicts shorter TTFT, even in the absence of palpable disease [71, 72]. Classical parameters such as LDT and serum β2‐M retain prognostic value, particularly when integrated into composite models [33, 73].

In summary, early‐stage CLL represents a spectrum of biologically distinct entities rather than a single clinical condition. Integrating IGHV status, BcR stereotypy, cytogenetics (including CK), recurrent mutations, surface markers, transcriptomic and serum biomarkers, imaging‐defined tumor burden, and classical clinical variables enables more accurate estimation of TTFT, which, in the near future, may improve patient counselling and rational design of trials exploring early or pre‐emptive interventions [6, 19].

## 3 Integrated Prognostic Models

The clinical course of early‐stage CLL is highly heterogeneous, and single markers alone—whether immunogenetic, cytogenetic, or biochemical—rarely capture the full spectrum of patient risk. Integrated prognostic models were therefore developed to combine multiple dimensions of disease biology, enabling more precise risk stratification and informing both monitoring intensity and therapeutic decision‐making [4, 6, 19, 74]. Among asymptomatic patients, these tools are particularly important for predicting TTFT, which represents the key clinically relevant endpoint in early‐stage disease.

### 3.1 CLL‐IPI and Its Validation in Early‐Stage CLL

Among available tools, the Chronic Lymphocytic Leukemia International Prognostic Index (CLL‐IPI) is the most widely validated model. CLL‐IPI integrates five independent variables—age, clinical stage (Rai or Binet), serum β2‐microglobulin, IGHV mutational status, and TP53 aberrations (deletion or mutation)—to stratify patients into four risk groups (low, intermediate, high, and very high) that correlate with both TTFT and OS across all stages of CLL [75]. In a prospective series of untreated Binet A patients, CLL‐IPI clearly discriminated therapy‐free survival strata, and subsequent external validations confirmed its robustness for TTFT prediction in early‐stage CLL [67, 68]. Importantly, CLL‐IPI captures the combined effect of molecular lesions and clinical variables; for example, a patient with unmutated IGHV and a subclonal TP53 mutation may be assigned to a higher‐risk category than clinical stage alone would suggest [75, 76, 77, 78]. Moreover, it is able to predict TTFT in patients whose only manifestation of disease is a circulating clonal lymphocyte population (monoclonal B‐cell lymphocytosis) [79].

In a comparative analysis of five prognostic systems (Rai and Binet staging, the MD Anderson Cancer Center—MDACC—score, the Barcelona score, and CLL‐IPI) focused on TTFT in early‐stage CLL, CLL‐IPI provided the most accurate prediction of TTFT, outperforming all other models [80]. These findings established CLL‐IPI as a benchmark reference for risk assessment in both unselected and early‐stage CLL populations.

### 3.2 MD Anderson Cancer Center (MDACC)‐Derived and Related Integrated Clinical–Biological Models

Several additional models—including MD Anderson Cancer Center (MDACC)‐derived nomograms, laboratory‐based scores, and biologically driven risk algorithms—have demonstrated strong predictive performance with extensive external validation. Italian multicenter studies confirmed the applicability of the MDACC nomogram and prognostic index in routine clinical practice and across diverse clinical settings [81, 82]. Across multiple analyses, models incorporating molecular and immunophenotypic variables have consistently outperformed those based solely on clinical parameters, particularly for TTFT prediction in early disease [83, 84].

Building on this framework, Molica et al. proposed a modified MDACC score that includes LDT, β2‐M, IGHV mutational status, and CD38 expression. This algorithm effectively stratified patients with clinical monoclonal B‐cell lymphocytosis (cMBL) and Rai stage 0 CLL into low‐, intermediate‐, and high‐risk groups for early treatment requirement, with high‐risk patients showing significantly shorter TTFT [85]. These findings underscore how the integration of biological and kinetic parameters into clinical indices improves risk stratification at the earliest disease stages.

A complementary approach has involved the development of strictly laboratory‐based scores focused on Rai stage 0 CLL. Cohen et al. used routinely available laboratory parameters—absolute lymphocyte count, β2‐M, IGHV mutation status, 17p and 11q deletion, and trisomy 12—to derive a weighted scoring system that was validated in an independent cohort. This model stratified Rai 0 patients into low‐, intermediate‐, and high‐risk groups for progression to treatment, with high‐risk patients demonstrating significantly shorter TTFT, supporting its use for closer surveillance and earlier identification of patients at increased risk of progression [86]. Similarly, our group showed that combining cellular and molecular markers such as CD38, ZAP‐70, and cytogenetic abnormalities identifies Binet A patients at increased risk of disease progression [87].

In parallel, the prospective O‐CLL1‐GISL study validated a biologically driven risk score incorporating IGHV status, ZAP‐70, CD38, and cytogenetic abnormalities for predicting progression‐free survival (PFS) in Binet A patients, further confirming the clinical value of biologically oriented models in early‐stage CLL [88]. Collectively, these integrated indices illustrate how the combination of clinical, biochemical, and genetic information substantially improves the prediction of disease progression and treatment requirement in early CLL.

### 3.3 IPS‐E and Simple Scores Tailored to Early‐Stage CLL

Because most patients with early‐stage CLL are asymptomatic, clinicians require a practical, TTFT‐focused prognostic tool that can be applied with minimal testing. The International Prognostic Score for Early‐stage CLL (IPS‐E) was specifically developed to address this need and was derived from a cohort of nearly 5000 previously untreated early‐stage patients. IPS‐E deliberately minimizes complexity by incorporating only three readily available parameters—unmutated IGHV, absolute lymphocyte count ≥ 15 × 10^9^/L, and palpable lymphadenopathy—yet reliably stratifies patients according to their 5‐year risk of treatment initiation [89]. Its simplicity, combined with robust external validation [90], makes IPS‐E particularly well suited for routine risk‐adapted monitoring and for identifying asymptomatic patients at increased short‐term risk who may be considered for enrollment into early‐treatment clinical trials.

### 3.4 The Central Role of Lymphocyte Doubling Time

The German CLL Study Group Score is another integrated model that incorporates clinical variables (age, Rai/Binet stage), laboratory parameters (β2‐M, thymidine kinase, LDT), molecular markers (IGHV status), and cytogenetic abnormalities. GCPI stratifies patients into low‐, intermediate‐, and high‐risk groups for disease progression and TTFT [91]. Importantly, external validation has demonstrated that GCPI retains prognostic value in early‐stage CLL even when thymidine kinase is unavailable, supporting its applicability in real‐world settings with limited laboratory resources [92].

The CLL1‐Prognostic Model (CLL1‐PM) was specifically designed for newly diagnosed Binet A patients, a population in which clinical stage no longer provides meaningful discrimination and in which the primary clinical question is the timing of treatment initiation rather than OS [93]. By excluding stage, the model focuses on variables that more directly reflect early disease aggressiveness. While CLL1‐PM shares some elements with CLL‐IPI—such as age, β2‐M, and IGHV status—it uniquely incorporates LDT and retains del(11q) and del(17p) as independent adverse factors. These features capture not only underlying disease biology but also growth kinetics, which are central to determining TTFT. LDT introduces temporal information that is absent from CLL‐IPI but is highly informative in early‐stage disease, where subtle changes in proliferation often precede clinical progression. As a result, the CLL1‐PM model demonstrates superior discrimination for TTFT in Binet A patients, whereas CLL‐IPI performs less optimally in this specific context because it was not calibrated to predict treatment initiation in early disease [93].

**FIGURE 2:**
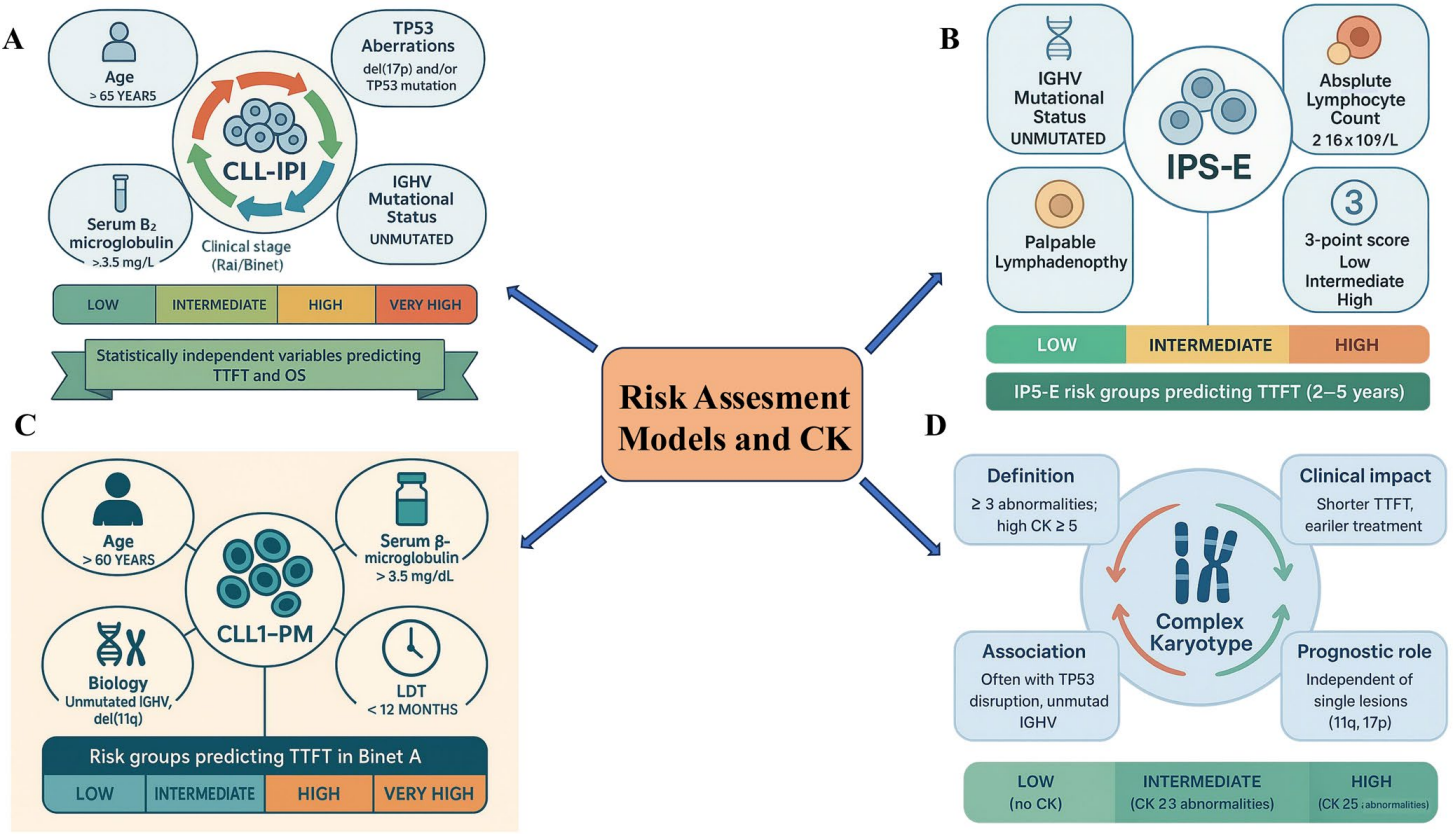
Integrated prognostic models in early‐stage CLL. Schematic overview of major prognostic tools used to predict time to first treatment and outcome in early‐stage CLL. Early indices such as the CLL‐IPI, developed for all disease stages and subsequently validated in asymptomatic/Binet A patients, combine age, clinical stage, β2‐microglobulin, IGHV status, and TP53 aberrations. More recent tools, including IPS‐E, integrated genetic–clinical models and purely laboratory‐based scores, progressively incorporate cytogenetic and mutational data, recurrent gene lesions, and transcriptomic/epigenetic features. Emerging multi‐omic and machine learning approaches represent the latest step in this evolution, aiming to provide individualized estimates of TTFT and progression risk.

The central role of LDT in TTFT prediction is further supported by independent analyses showing that models incorporating LDT consistently outperform those excluding it. Across the O‐CLL1 training cohort and the Novara and Barcelona validation sets, inclusion of LDT was associated with higher Harrell's C indices, greater explained variance in TTFT, and strong support by Akaike weights, whereas models omitting LDT showed negligible statistical support [33]. Notably, LDT provides prognostic information independent of established risk factors, including IGHV mutational status, del(11q), del(17p), β2‐M, Rai stage I–II, and NOTCH1 mutations; its exclusion resulted in a clinically meaningful loss of predictive accuracy [33]. An overview of the principal integrated prognostic tools, their core variables, and target endpoints is shown in Figure 2. Collectively, these models exemplify the evolution of prognostication in early‐stage CLL—from purely clinical indices toward composite scores integrating biochemical, immunogenetic, and cytogenetic parameters, and more recently toward multi‐omic and machine learning–based approaches. This progression has substantially improved our ability to stratify asymptomatic patients by TTFT and progression risk, enabling more refined risk‐adapted monitoring and the identification of candidates for early‐intervention clinical trials.

### 3.5 Emerging Genomic, Multi‐Omic, and Machine‐Learning Models

Beyond established prognostic indices, more recent research highlights the added value of integrating genomic complexity, recurrent somatic mutations, and clonal evolution with clinical variables [94, 95]. Incorporating cytogenetic and mutational data—such as recurrent mutations in NOTCH1, SF3B1, ATM, and BIRC3—alongside biochemical and clinical markers improves predictive accuracy, especially for identifying high‐risk patients who may progress despite favourable anatomical staging. In parallel, emerging multi‐omic and machine‐learning approaches, including deep learning and explainable artificial intelligence, aim to further refine individualized risk predictions. These strategies integrate heterogeneous data layers—genomic, transcriptomic, and clinical—to generate dynamic risk estimates and may ultimately support precision surveillance and pre‐emptive therapeutic strategies in early‐stage CLL [94, 95].

Looking ahead, the next generation of TTFT prediction is likely to unify static clinical and immunogenetic markers, baseline genomic complexity, and dynamic disease signatures—such as clonal evolution, transcriptomic shifts—within machine‐learning frameworks trained on large, harmonized cohorts [4, 6, 12, 74, 75, 78, 87, 92, 93]. Such hybrid models could enable more precise, individualized forecasts of treatment need and, critically, restrict early‐intervention trials to those asymptomatic patients whose biology clearly portends near‐term progression. Until these tools are fully mature and externally validated, however, the safest and most evidence‐based strategy remains risk‐adapted watch‐and‐wait, guided by validated prognostic indices. This approach preserves therapy for patients who demonstrably require treatment while focusing research efforts on the development and validation of next‐generation prognostic algorithms.

### 3.6 Clinical Implications for Early Treatment Strategies

Prognostic models for early‐stage CLL—whether based purely on biological variables, integrated clinical–biological indices, or scores explicitly incorporating LDT—are crucial for moving beyond a uniform watch‐and‐wait paradigm toward a more selective, biology‐driven approach to early intervention. By integrating markers such as IGHV mutational status, del(11q) and del(17p), β2‐M, Rai/Binet stage, LDT, and recurrent gene mutations (e.g., NOTCH1), these tools can reliably distinguish patients whose disease is likely to remain indolent for many years from those with a high probability of requiring treatment in the near term despite not yet fulfilling standard iwCLL criteria [5]. In clinical practice, this stratification enables low‐risk patients to be confidently managed with conventional surveillance, sparing them unnecessary therapy and reducing the overall healthcare burden. This approach is further supported by the fact that disease progression rarely occurs in a rapid or catastrophic fashion, allowing most patients to be safely monitored. Moreover, in the current therapeutic era the implications of delaying or deferring systemic treatment may be less significant, as targeted agents are becoming the standard frontline option rather than chemoimmunotherapy. Conversely, high‐risk patients can be systematically identified and prioritized for closer monitoring and/or enrollment in early‐intervention trials evaluating novel targeted therapies or risk‐adapted treatment strategies. Across validated models, the central value lies not only in prognostication but also in providing an objective framework for enriching clinical studies with early‐stage CLL patients most likely to benefit from experimental early treatment approaches.

## 4 Historical Perspectives on Early Treatment

The concept of early intervention in CLL has been explored for decades, dating back to the era when chemotherapy was the primary treatment option. Early trials in the 1980s and 1990s investigated whether initiating therapy at diagnosis—rather than deferring therapy until clinical progression—could improve OS [96, 97, 98, 99, 100, 101, 102, 103] (Table 1). A phase 3 study conducted on a small cohort of Binet stage A CLL patients demonstrated that interferon alfa did not provide benefit in terms of PFS and OS [91]. Agents such as chlorambucil, cyclophosphamide, and later fludarabine were evaluated in randomized and prospective studies that predominantly enrolled asymptomatic patients with early‐stage disease (Rai 0–II or Binet A–B) [97, 98, 99, 100, 101, 102, 103].

**TABLE 1:** Historical early‐treatment trials in CLL.

Trial/group	Drug(s) tested	Patient population	Primary endpoint	Key findings	References
Langenmayer et al.	Interferon‐alpha 2b	Early‐stage, asymptomatic CLL	Overall survival (OS) and progression‐free survival (PFS)	Median PFS: Interferon‐α2b: 6.7 month High‐risk control: 13.3 month Low‐risk observational group: 37 month Median OS: Interferon‐α2b: 44.9 month High‐risk control: 43.1 month Low‐risk group: 57.9 month No OS and PFS benefit from early treatment	[95]
Shustik et al.	Chlorambucil	Binet A, early‐stage CLL	OS	5‐year OS: Chlorambucil: ~75% Observation: ~75% No significant difference in OS between patients treated immediately with chlorambucil and those managed with a deferred (watch‐and‐wait) strategy.	[96]
German CLL1	Fludarabine	Binet A, early‐stage CLL, high‐risk	OS, time to first treatment (TTFT)	Median PFS: 59.4 month Median OS: not reached. 10‐year OS ≈74.7%. Median TTFT (TTST): 110.2 months Early fludarabine improved TTFT, but no OS benefit	[97]
CLL trialists' meta‐analysis	Chlorambucil/Fludarabine	Early‐stage CLL (multiple RCTs)	OS	Early‐stage CLL (immediate vs. deferred chemotherapy) 10‐year OS Immediate chemotherapy: 44% Deferred chemotherapy: 47% Advanced disease (combination chemotherapy vs. chlorambucil) 5‐year OS: 48% in both groups Early treatment did not improve long‐term survival	[102]
Hoechstetter et al.	Intermittent chlorambucil	Early‐stage/indolent CLL	OS, toxicity	No survival advantage; higher rates of hematologic toxicity with early therapy	[92]
French cooperative group	Chlorambucil	Binet stage A, low‐risk	OS, progression‐free survival (PFS)	Relative risk of death: Trial 1: RR = 1.14 (95% CI 0.92–1.41; *p* = 0.23) Trial 2: RR = 0.96 (95% CI 0.75–1.23; *p* = 0.74) No improvement in OS; minor improvement in PFS	[98]
CLL7 trial (German‐french)	FCR (Fludarabine + Cyclophosphamide + Rituximab)	Binet A high‐risk	OS, event‐free survival (EFS)	Median EFS: Early FCR: Not reached Watch‐and‐wait: 18.5 months 5‐year EFS rate: Early FCR: 55.2% Watch‐and‐wait: 12.6% 5‐year OS: Early FCR: 82.9% Watch‐and‐wait: 79.9% Higher EFS with early FCR, no OS improvement	[104]

These trials consistently demonstrated that early treatment with conventional chemotherapy did not confer a survival advantage. Long‐term follow‐up from the IGCI and German CLL1 studies showed that initiating chlorambucil or fludarabine in asymptomatic patients failed to improve OS compared to the watch‐and‐wait strategy, despite transient reductions in lymphocyte counts or lymphadenopathy [98, 101].

Meta‐analyses pooling data from multiple randomized trials further confirmed that early cytotoxic therapy did not prolong survival [103].

A critical lesson from these studies is that early chemotherapy could compromise the effectiveness of subsequent treatments. Patients receiving front‐line cytotoxic therapy frequently experienced cumulative toxicity, myelosuppression, and an increased risk of therapy‐resistant disease clones, thereby limiting options for later‐line interventions [97, 98, 99, 100, 101, 102, 103, 104]. Furthermore, these trials highlighted the natural history of early‐stage CLL, demonstrating that many patients remain clinically stable for years or even decades without requiring therapy [97, 98, 99, 100, 101, 102, 103, 104].

As a result, the watch‐and‐wait strategy emerged as the standard of care for asymptomatic patients with early‐stage disease. This approach prioritizes monitoring and timely intervention only when clear indications—such as clinical progression, cytopenias, or symptomatic organ involvement—develop [90, 91, 92, 93, 94, 95, 96, 97]. Importantly, this paradigm reflects a balance between minimizing treatment‐related harm and ensuring that patients are treated before irreversible complications occur.

Beyond clinical outcomes, these early trials also underscored the profound biological heterogeneity of CLL, which was not captured by anatomical staging alone. Patients with favourable prognostic features [e.g., mutated IGHV, or isolated del(13q14)] often remained stable for prolonged periods, whereas those with high‐risk characteristics (e.g., unmutated IGHV, TP53 abnormalities) progressed more rapidly. Although such molecular insights were not available at the time these trials were conducted, retrospective analyses have shown that the lack of OS benefit from early chemotherapy was consistent across most biological subgroups, further validating watch‐and‐wait as a safe, evidence‐based standard [97, 98, 99, 100, 101, 102, 103, 104].

The German CLL7 trial later evaluated whether early treatment with FCR chemoimmunotherapy could improve outcomes in patients with early‐stage (Binet A) harboring high‐risk cytogenetic features. The investigators demonstrated that FCR significantly prolonged event‐free survival and delayed disease progression compared with watch‐and‐wait. However, this benefit did not translate into improved OS, and treatment‐related toxicity was substantial. Importantly, this study showed for the first time in a genetically defined high‐risk early CLL population that early intensive chemoimmunotherapy still fails to improve survival, reinforcing watch‐and‐wait as the standard approach even in patients with unfavorable prognostic features [105].

### 4.1 Early Intervention in the Targeted Therapy Era

The advent of targeted therapies has fundamentally changed the treatment landscape of CLL, particularly for patients with high‐risk disease. Unlike conventional chemotherapy, targeted agents such as BTK inhibitors (e.g., ibrutinib) and BCL2 inhibitors (e.g., venetoclax) act on specific signaling pathways critical for CLL cell survival and proliferation [6]. These therapies have demonstrated the ability to induce deep and durable responses, even in molecularly high‐risk subsets, thereby renewing interest in the concept of early intervention in biologically high‐risk, asymptomatic patients [6]. In the current therapeutic era, targeted agents are becoming the standard frontline option rather than chemoimmunotherapy, potentially altering the risk–benefit ratio of early intervention [106].

Ibrutinib, a first‐in‐class BTK inhibitor, irreversibly blocks B‐cell receptor signalling, disrupting proliferation and survival signals in CLL cells. The CLL12 trial, a randomized study evaluating early versus delayed ibrutinib in patients with high‐risk early‐stage CLL, demonstrated a significant improvement in event‐free survival (EFS) for early treatment, but no OS benefit was observed after a median follow‐up of 31 months [107, 108]. Although ibrutinib was effective in delaying disease progression, cardiovascular adverse events—including atrial fibrillation, hypertension, and bleeding complications—were non‐negligible and tempered enthusiasm for its broad application in asymptomatic patients [107, 108]. These findings emphasize that even targeted therapies, despite improved efficacy and tolerability compared with chemotherapy, are not devoid of clinically relevant toxicity, reinforcing the need for careful patient selection.

Several phase II studies are currently exploring the role of novel agents as early intervention strategies in asymptomatic, early‐stage CLL, with a particular focus on BTKis, either as monotherapy or in combination regimens. A randomized phase II study conducted at The Ohio State University enrolled 44 patients with biologically high‐risk disease—defined by unmutated IGHV status, high‐risk FISH abnormalities, or complex karyotype—and assigned them to receive ibrutinib either concurrently with or sequentially following vaccination with PCV13, trivalent influenza, and DTaP vaccines. Early treatment with ibrutinib was feasible and generally well tolerated, with no grade 4 toxicities and no grade 3–4 hematologic adverse events; two patients developed grade 3 atrial fibrillation. Notably, early intervention was associated with improvements in patient‐reported QoL measures, particularly cancer‐related stress, anxiety, and sleep disturbance [107, 108]. In this regard, a revision of diagnostic criteria and a reconsideration of when a diagnosis should be made, balancing scientific evidence with patient wellbeing has been proposed in order to reduce the medicalization of harmless conditions [109]. In parallel, four additional phase II studies have been conducted in patients with high‐risk, asymptomatic early‐stage CLL, evaluating ibrutinib either alone [107, 108, 109, 110] or in combination with pembrolizumab [111], as well as acalabrutinib, either as monotherapy or combined with obinutuzumab [112, 113, 114]. To date, none of these studies has reported an OS benefit. Another ongoing randomized study, Prevent‐ACaLL (NCT03868722), is enrolling 212 patients at increased risk of infection and/or treatment requirement, as defined by the CLL‐TIM algorithm, and is evaluating a fixed‐duration, 12‐week course of acalabrutinib plus venetoclax versus placebo. The primary endpoint is survival free from grade ≥ 3 infections at 24 weeks, corresponding to 12 weeks after completion of study treatment [115].

Venetoclax, a selective BCL2 inhibitor, induces apoptosis in CLL cells by antagonizing anti‐apoptotic BCL2 proteins. Venetoclax‐based regimens—especially in combination with anti‐CD20 monoclonal antibodies such as obinutuzumab—have achieved deep responses with high rates of undetectable MRD in both frontline and relapsed/refractory settings [116, 117, 118]. The EVOLVE trial is a randomized phase III study comparing early treatment with venetoclax plus obinutuzumab versus delayed treatment in asymptomatic, high‐risk patients defined by CLL‐IPI ≥ 4 and/or complex cytogenetics. Early results suggest that initiating venetoclax‐based therapy before clinical indications may improve PFS and increase deep remission rates compared with a watch‐and‐wait strategy; however, longer follow‐up is required to determine the impact on long‐term outcomes, including OS [119].

Finally, in a phase II trial with extended follow‐up, early intervention with lenalidomide in asymptomatic patients with high‐risk CLL resulted in durable disease control and a prolonged time to next therapy, with an estimated 8‐year overall survival of nearly 75%. Rates of grade ≥ 3 infections and second neoplasms were consistent with those expected in this high‐risk population, supporting the long‐term feasibility of this approach [120]. Table 2 describes early‐intervention trials with targeted therapy in CLL.

**TABLE 2:** Early‐intervention trials with targeted therapy in CLL.

Trial	Drug(s)	Patient population	Primary endpoint	Key findings	References
CLL12	Ibrutinib	High‐risk, early‐stage CLL (treatment‐naïve)	Event‐free survival (EFS), OS	Median EFS: Ibrutinib: NR Placebo: 51.6 month Median PFS: Ibrutinib: NR Placebo: 14 month Median TTNT: Ibrutinib: NR Placebo: 68.5 month 5‐year OS: Ibrutinib: 93.3% Placebo: 93.6% Watch‐and‐wait cohort: 97.9% Improved EFS with early ibrutinib, no OS benefit; cardiovascular toxicity notable	[105, 106]
NCT03207555	Ibrutinib	Untreated CLL and estimated TTFT of 3 years or less according to the MDACC nomogram	CR, CRi	There are no reported clinical outcomes	[109]
NCT02518555	Ibrutinib concurrently with vaccination (PCV13, trivalent influenza, and DTaP) or sequentially after vaccination	Untreated, asymptomatic CLL/SLL and ≥ 1 high‐risk genomic features: del(17p13.1), del(11q22.3), Complex karyotype (≥ 3 cytogenetic abnormalities on stimulated karyotype) unmutated IGHV Zap‐70 > 20%	2‐year PFS, MRD	There are no reported clinical outcomes	[108]
NCT03514017	Pembrolizumab in combination with ibrutinib	High‐risk patients with untreated CLL	ORR, TTR, PFS	There are no reported clinical outcomes	[110]
NCT04178798	Acalabrutinib or observation	Untreated asymptomatic CLL with GCLLSG prognostic index with intermediate [3, 4, 5], high [6, 7, 8, 9, 10], or very high [11, 12, 13, 14] risk scores	EFS, PFS, OS, ORR, TTNT	There are no reported clinical outcomes	[112]
NCT03516617	Acalabrutinib vs. acalabrutinib and obinutuzumab	Untreated CLL/SLL and high‐or very high‐risk CLL‐IPI score randomized into 2 treatment arms. Low‐intermediate CLL‐IPI to be observed	Rate of MRD‐, CR (arm A and arm B). TTFT (arm C)	There are no reported clinical outcomes	[111]
Prevent‐aCaLL	Short‐term combined acalabrutinib and venetoclax treatment	CLL at high risk of infection and/or progressive treatment within 2 years according to CLL‐Tim	Rate of serious infections during the treatment period	There are no reported clinical outcomes	[114]
EVOLVE (SWOG)	Venetoclax and obinutuzumab or delayed therapy with venetoclax and obinutuzumab	Untreated asymptomatic CLL or SLL High risk CLL‐IPI score ≥ 4 Complex cytogenetics (≥ 3 cytogenetic abnormalities)	OS at 6 years, FACT	There are no reported clinical outcomes	[118]
NCT01351896	Concurrent lenalidomide and PCV13 or sequential PCV14 and lenalidomide	Adult patients with untreated, asymptomatic CLL/SLL and ≥ 1 high‐risk genomic features: del17p; del11q; Complex karyotype (≥ 3 cytogenetic abnormalities); unmutated IGHV	OS, PFS, TTNT	There are no reported clinical outcomes	[119]

## 5 Controversies and Limitations

**FIGURE 3:**
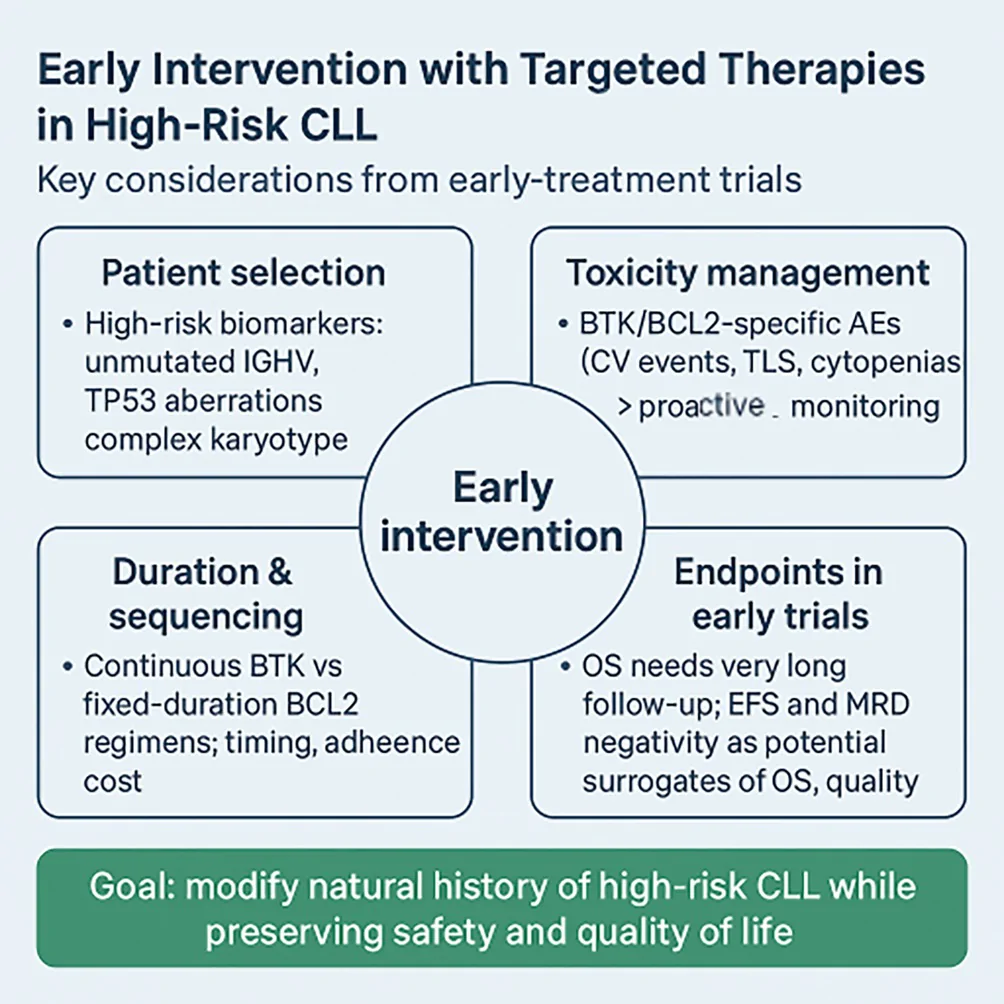
Key considerations for early intervention with targeted therapies in high‐risk CLL.Schematic overview of the main domains influencing the use of early targeted therapy in asymptomatic, high‐risk CLL. At the center, “Early intervention” is surrounded by four interconnected components. Patient selection highlights the role of high‐risk biomarkers—such as unmutated IGHV, TP53 aberrations, and complex karyotype—in identifying candidates most likely to benefit from early treatment. Toxicity management summarizes class‐specific adverse events associated with BTK and BCL2 inhibitors (e.g., cardiovascular events, tumour lysis syndrome, cytopenias), emphasizing the need for structured monitoring and supportive care. Duration and sequencing contrasts continuous BTK inhibitor therapy with fixed‐duration venetoclax‐based regimens, raising issues of optimal timing, adherence, and cost‐effectiveness. Endpoints in early‐intervention trials indicate that, given the long natural history of early‐stage CLL, overall survival is complemented by surrogate measures such as event‐free survival, MRD negativity, and quality of life. Collectively, these elements illustrate how biological risk, safety profile, treatment strategy, and trial endpoint selection must be integrated when designing and interpreting early‐treatment approaches in CLL.

Despite growing interest, early intervention in CLL, particularly with targeted agents, remains associated with substantial uncertainties that currently preclude its routine use in clinical practice. Evidence from early‐treatment trials have highlighted four main areas of concern (Figure 3). First, patient selection remains imperfect. Biological features—such as unmutated IGHV, TP53 aberrations, and complex karyotype—are commonly used to identify patients most likely to benefit from early treatment [10, 11, 17, 18, 19, 20], but their correct definition for clinical purposes, for example, in the case of TP53 mutations or complex karyotype, remain elusive as for the mutation cut‐off or the number of cytogenetic aberrations to calculate [19, 44, 45, 46]. In addition, these markers do not fully capture the biological and clinical heterogeneity of early‐stage CLL. Importantly, a significant proportion of patients classified as “high‐risk” patients may still experience prolonged treatment‐free survival, raising concerns about misclassification and unnecessary exposure to therapy.

Second, toxicity and overtreatment represent non‐trivial risks. Although BTK and BCL2 inhibitors are generally better tolerated than chemoimmunotherapy, they are associated with distinct adverse‐event profiles—including cardiovascular complications, tumor lysis syndrome, and cytopenias—that require structured monitoring and proactive management [121]. Administering continuous or fixed‐duration therapy to patients who might never progress exposes them to cumulative toxicity and potential drug resistance in the absence of a proven survival advantage and may compromise tolerance of, or eligibility for, future lines of treatment [117].

Third, the therapeutic paradigm itself raises important practical questions. Unlike fixed‐course chemotherapy, targeted therapies may be delivered as continuous BTK inhibition or as time‐limited venetoclax‐based combinations. In an otherwise asymptomatic population, the optimal timing of initiation, duration of therapy, and sequencing remain undefined, with significant implications for treatment adherence, QoL, and cost‐effectiveness [117]. The high cost of these agents—often administered for prolonged periods—further challenges the justification for early use in the absence of a clear OS benefit [122]. In this context, the economic companion study NCT05371808 is investigating differences in healthcare utilization and cost between early and delayed treatment strategies in asymptomatic, high‐risk CLL/SLL patients; results are currently pending [123].

Fourth, important methodological limitations affect trial endpoints. Most early‐intervention studies rely on surrogate measures—such as EFS or PFS and depth of response, including MRD negativity—rather than OS or long‐term QoL [106, 107, 119]. While these endpoints reflect disease control, their validity as surrogates in asymptomatic early‐stage CLL—where the natural history is highly heterogeneous and often spans decades—remains unproven. Consequently, it is unclear whether improvements in EFS or MRD negativity translate into outcomes that are clinically meaningful for patients who might otherwise remain stable for many years.

Taken together, these issues—imperfect patient selection, the risk of overtreatment and toxicity, economic burden, and reliance on surrogate endpoints—underline that early intervention with targeted therapies, while biologically and conceptually attractive, should still be considered experimental. At present, watch‐and‐wait with risk‐adapted monitoring remains the standard of care, and early therapy should be confined to well‐designed clinical trials. Careful patient counselling and individualized decision‐making, integrating molecular risk, comorbidities, patient preferences, and emerging evidence, are essential while ongoing studies continue to test whether early intervention can deliver durable, clinically meaningful benefit in high‐risk CLL [106, 107, 119].

## 6 Conclusions

Early‐stage CLL is a clinically and biologically heterogeneous condition. Even with modern genomic profiling, cytogenetics, and longitudinal assessment of clonal evolution, its natural history remains highly variable: many patients remain stable for years or decades, whereas a minority progress relatively rapidly. Moreover, there is no early intervention known to reduce the risk of Richter transformation. Therefore, further research could focus particularly on the subgroup of patients at high risk for this feared complication. Watch‐and‐wait remains the recommended strategy for asymptomatic patients, offering the dual advantages of avoiding unnecessary toxicity and preserving future therapeutic options [5]. Tailored follow‐up, guided by established prognostic models, enables timely identification of progression without exposing patients to the risks associated with premature treatment [5, 6].

Targeted agents—including BTK inhibitors, BCL2 antagonists, and next‐generation anti‐CD20 antibodies—have transformed the outlook of symptomatic CLL, achieving deep remissions and high rates of MRD negativity even in biologically high‐risk subsets [116, 117, 118, 119, 120]. These advances have naturally fueled interest in early‐intervention strategies for asymptomatic but high‐risk patients. Trials such as CLL12 and EVOLVE show that early use of targeted agents can improve EFS, modify the natural history of immune dysfunction, and increase MRD clearance; however, none have yet demonstrated a clear OS advantage [106, 107, 110, 119]. This is partly because OS in early‐stage CLL is fortunately very long, making survival advantages difficult to demonstrate within the time frame of current studies, particularly as increasingly effective therapies remain available at the time of progression. Furthermore, the advanced median age of patients at diagnosis—typically around 72 years—demands additional caution, as the potential for treatment‐related toxicities may more easily outweigh the benefits in a population often managed for multiple comorbidities. At the same time, targeted agents are associated with specific toxicities, often require prolonged or repeated exposure, and impose substantial economic costs, all of which heighten the risk of overtreatment in patients who might otherwise enjoy many years without therapy [106, 107, 119, 124].

More refined biological stratification may eventually enable a more selective, biology‐driven approach to early treatment. In this perspective, a deeper understanding of the biological landscape is essential; for instance, dissecting clonal hematopoiesis within the myeloid compartment of CLL patients may provide crucial insights into disease stability and the mechanisms driving Richter transformation [125, 126]. Integrating information on genomic instability, recurrent mutations, and dynamic biomarkers could help identify the small subset of early‐stage patients whose disease follows a truly aggressive trajectory and who might benefit from MRD‐guided or time‐limited early intervention [6, 8, 20].

## Author Contributions

All authors contributed to the manuscript and were involved in revisions and proofreading. All authors approved the submitted version.

## Funding

The authors have nothing to report.

## Conflicts of Interest

The authors declare no conflicts of interest.

## Data Availability

Data sharing not applicable to this article as no datasets were generated or analysed during the current study.
